# Agency of Subjects and Eye Movements in Schizophrenia Spectrum Disorders

**DOI:** 10.1007/s10936-022-09903-6

**Published:** 2022-07-16

**Authors:** Chiara Barattieri di San Pietro, Giovanni de Girolamo, Claudio Luzzatti, Marco Marelli

**Affiliations:** 1 Department of Psychology, University of Milano-Bicocca, Piazza dell’Ateneo Nuovo 1, 20126 Milan, Italy; 2 Psychiatric Epidemiology and Evaluation Unit, IRCCS Istituto Centro San Giovanni di Dio Fatebenefratelli, Brescia, Italy; 3 Milan Center for Neuroscience, Milan, Italy

**Keywords:** Schizophrenia spectrum disorders, Eye movements, Verb thematic roles, Language

## Abstract

People with schizophrenia spectrum disorders (SSD) show anomalies in language processing with respect to “who is doing what” in an action. This linguistic behavior is suggestive of an atypical representation of the formal concepts of “Agent” in the lexical representation of a verb, i.e., its thematic grid. To test this hypothesis, we administered a silent-reading task with sentences including a semantic violation of the animacy trait of the grammatical subject to 30 people with SSD and 30 healthy control participants (HCs). When the anomalous grammatical subject was the Agent of the event, a significant increase of Gaze Duration was observed in HCs, but not in SSDs. Conversely, when the anomalous subject was a Theme, SSDs displayed an increased probability of go-back movements, unlike HCs. These results are suggestive of a higher tolerability for anomalous Agents in SSD compared to the normal population. The fact that SSD participants did not show a similar tolerability for anomalous Themes rules out the issue of an attention deficit. We suggest that general communication abilities in SSD might benefit from explicit training on deep linguistic structures.

**Supplementary Information:**

The online version contains supplementary material available at 10.1007/s10936-022-09903-6.

## Introduction

Language anomalies are pervasive among people with schizophrenia spectrum disorders (SSD) (DeLisi, 2001), and disorganized speech (conceptualized as “formal thought disorder”) is one of the core diagnostic features of schizophrenia as indicated both by the DSM-5 (APA, 2013) and the ICD-10 (WHO, 1992). In the past decades, evidence has been cumulating towards a scenario in which all levels of language are impaired in SSD (Bellani et al., 2009), from phonetics (Stein, 1993) to pragmatics (Covington et al., 2005). To explain the anomalous language performance in SSD, a fundamental disorder of the semantic system has been hypothesized (Goldberg et al., 1998; Paulsen et al., 1996; Rossell & David, 2006), alongside with syntactic processing deficits (Condray et al, 2002; Tan & Rossell, 2019), affective problems (Minor et al, 2016), and referential anomalies (Çokal et al., 2018).

In schizophrenia, the presence of disorganized speech is generally reported as a “positive symptom” (i.e., the abnormal emergence of thoughts and behaviors otherwise absent in healthy individuals) and is mostly known as “Formal Thought Disorder” (FTD – American Psychiatry Association; 2013). Despite associations between FTD and language disturbances in SSD have been consistently reported (Little et al., 2019; Tan & Rossell, 2019), the nature of this relationship is still debated (Little et al., 2019). On the one hand, some studies have identified the root of impaired thinking in language deficits (Hinzen & Rosselló, 2015; Tan & Rossell, 2019), supporting this stance with evidence of neurological alterations of the language network (Horn et al., 2009; Li et al., 2007; Ratnanather et al., 2013; Sommer et al., 2003; Spaniel et al., 2007; Weiss et al., 2006). On the other, some studies have regarded these language deficits as the result of a reduction of general cognitive resources (e.g., Gold et al., 2002; Lelekov et al., 2000) and part of a broader cognitive impairment (Harrow et al., 2003; Oh et al., 2002; Kuperberg et al., 2010; Rodriguez-Ferrera et al., 2001; Tan & Rossell, 2015). This latter view has been substantiated by the fact that language disturbances in individuals with FTD appear early on and persist even when psychotic symptoms improve (Tan & Rossell, 2015).

Previous studies reported an underperforming word retrieval (Covington et al., 2005) as well as defective processing of words coming from high lexical competitor environments (high density and high-frequency lexical neighborhoods) (Titone & Levy, 2004) among people with schizophrenia. In particular, studies on verb naming seem to converge towards a significant impairment of action fluency (i.e., the ability to retrieve and produce action-related verbs) in this clinical population (Badcock et al., 2011; Kambanaros et al., 2010; Marvel et al., 2004; Smirnova et al., 2017; Woods et al., 2007). Given that verb lexical representations (or their “lemma”, that is, the abstract grammatical representation of words according to Levelt et al. 1999) contain information on “who is doing what” in the action described (i.e., the argument structure of the verb and its thematic grid), such impairment of action fluency may indicate a selective damage in encoding/decoding verb lexical representations and, more specifically, in the attribution of verb “agency” (Jeannerod, 2009).

However, when considered in the context of a sentence, verb processing entails both semantic and syntactic processing, and previous research in this clinical population identified a relative insensitivity to linguistic violations requiring the contribution of both syntax and semantics (Kuperberg et al., 1998). When listening to oral sentences that were rendered anomalous by pragmatic, semantic, or syntactic violations, results indicated that healthy controls and patients with schizophrenia but without thought disorder took longer to recognize target words (in this case, the direct object of the verb) preceded by linguistic anomalies, compared with words in normal sentences. On the contrary, thought-disordered patients showed significantly smaller differences in reaction times, suggestive of relative insensitivity to linguistic violations. Thought disorder in schizophrenia has been also linked to suboptimal use of linguistic context to process and produce speech. In a self-paced reading task (Kuperberg et al., 2006a), people with schizophrenia showed shorter reaction times than controls to sentence-final words when the Agent subject of the sentence was replaced with an inanimate noun, making the sentence implausible (e.g. “For breakfast the *eggs would only eat toast and jam”), as well as in the case of morpho-syntactically violated sentences (e.g. “For breakfast the boys would only *eats toast and jam”)^1^. Such relative insensitivity to these violations was interpreted as suggestive of a specific difficulty, in patients with schizophrenia, in combining semantic and syntactic information to build the linguistic context necessary to interpret the sentence. Electrophysiological evidence (Kuperberg et al., 2010) further suggests that, when studying the processes underlying the building up of sentence and discourse structure, it is not possible to separate, on the one hand, the structure and function of semantic memory and, on the other hand, the ability to combine and integrate words. Rather, language impairment in schizophrenia would result from a dysfunctional interaction between these systems to build up to higher-order meanings.

It must be noted, however, that the differential impact of different thematic roles in the preverbal subject position has not been tested in any of these studies. The understanding of “who is doing what to whom” in an action event is called “Thematic Role knowledge” (Wu et al., 2007), and the function that each participant plays in the action described by the verb is stored in the so-called “Thematic Grid” of the verb (Grimshaw, 1990). Central to our hypothesis is the notion of “thematic structure” of verbs, which has been fundamental in formal linguistics in the past 50 years (Fillmore, 1968; Gruber, 1965; Jackendoff, 1972). This model stems from the concept of “linguistic universal” (Chomsky, 1965) or the idea that some linguistic patterns occur systematically across all natural languages. The validity of the model has been repeatedly confirmed (for Italian see: Belletti & Rizzi, 1988; Graffi, 1994; Vernice & Sorace, 2018), and has been adopted as a framework to study verb acquisition in children (Fisher, 2002), as well as pathological language deficits (for example, in aphasia see Barbieri et al., 2010; Bazzini et al., 2012; Caramazza et al., 1991). In the action event, the participant who causes something to happen is generally understood to occupy the thematic role of “Agent”, while the “Theme” is the entity passively involved in the event expressed in the predicate. To our knowledge, no studies have considered whether, and how, the so-called “disorder of the self”, whose presence has been theorized in SSD population (Sass & Parnas, 2003), might come into play in this context, considering that the notion of “agency” is usually conveyed by the thematic role of “Agent” stored in the lexical representation (the thematic grid) of the verb. According to the Self-Disorder theory, the basic experience of being a self appears to be unstable in schizophrenia, resulting in a markedly diminished sense of “mine-ness” of one’s thoughts, body, and actions (Henriksen & Noordgard, 2014). Studies investigating the association between Self Disorder and neurocognitive dysfunction in the SSD population found that high levels of Self Disorder were associated with verbal memory impairments, but not with other neurocognitive functions (Haug et al., 2012). Thus, the ability to process incoming verbal information and organize it efficiently concerning pre-existing self-knowledge appears to be suboptimal in the SSD population, possibly hindering the ability to comprehend, direct, remember, and reason about one’s thoughts and self-knowledge, which are functions known to be related to several aspects of Self Disorder. Abnormalities in agency inference have been observed in SSD patients (Prikken et al., 2018), and research on misattribution of agency in schizophrenia yielded to identify an imprecise internal prediction about sensory consequences of one’s action in this population (Synofzik et al., 2010).

A complex scenario emerges, in which psychopathology and syntactic-semantic processing seem to intertwine for language processing in people with SSD. Eye-tracking techniques can provide the opportunity to collect quantitative measures that are thought to mirror moment-to-moment cognitive processes demands (Rayner, 1998) during text comprehension, providing information on the temporal order of such processes. The assumption behind eye-tracking techniques is that a portion of text containing a semantic incongruence disrupts text comprehension, hence increasing processing demands. This happens, for example, when semantic and/or syntactic expectations are not met, requiring the reader to re-process the text to figure out the actual incongruence. This reprocessing is generally realized by means of a larger number of regressive movements (re-reading the same or previous words) or longer reading time associated with the portion of text characterized by most substantial processing demands. It has been demonstrated that readers tend to fixate longer those regions of the text that are the locus of inconsistency, and that, in these cases, the probability of making a regressive eye movement increases as well (Rayner et al., 2006). Early studies on eye-movement during verb processing showed that, instead of immediately adopting a meaning based on context and frequency information, people tend to delay the interpretation of verbs and resolve them considering subsequent as well as preceding context (Pickering & Frisson, 2001).

Previous studies analyzing eye movements in schizophrenia during text reading (sentences with canonical word order) found robust oculomotor markers of reading difficulty in the form of reduced forward saccade amplitude (Whitford et al., 2013), slower reading rates, increased number of saccades paired with decreased saccadic amplitude (Roberts et al., 2013), fewer single fixations, more second pass fixations, and increased gaze duration (Fernández et al., 2016) than in matched controls. These differences have been interpreted as suggestive of differences in high-order context processing (Whitford et al., 2018) as well as lower-level motor and perceptual functions. However, to the best of our knowledge, eye movements of individuals with SSD when reading anomalous texts (i.e., semantic violations) have not been investigated before. In this sense, we believe that a better characterization of the cognitive processes underlying Thematic Role comprehension might shed some light on the link between Self Disorder and language in this clinical population.

The present study aimed to assess eye-movement patterns in people with SSD when reading sentences with semantic violations affecting the “animacy” trait of different thematic roles (i.e., Agent *vs.* Theme). Given the presence of Self Disorder (Henriksen & Noordgard, 2014) and based on previous results pointing to a specific tolerance to semantic violations involving verb processing (Kuperberg et al., 2006a), we expected people with SSD to show increased tolerance to semantic violations affecting the animacy trait of the Agent. In other words, when processing sentences with animacy violations on the Agent subject, we do not expect people with SSD to display signs of an implicit detection of an anomaly. We expect this tolerance to be visible in eye-tracking indices of access to the lexical properties of the verb (Bock & Levelt, 1994).

## Material and Methods

### Participants

Thirty participants with a diagnosis of SSD according to DSM-5 (APA, 2013) were recruited. Participants were aged 18–65, able to give informed consent, and right-handed, and had normal or corrected-to-normal visual acuity. Diagnoses were made by the treating clinician (staff psychiatrist). Co-morbidities with personality and developmental disorders were also admitted. Exclusion criteria were: neurological disorders, head trauma with cognitive sequelae, intellectual disability, substance abuse in the 3 months preceding the enrollment. At recruitment, patients had been on treatment with at least one antipsychotic medication for at least the previous 6 months. Mean illness duration for the SSD group was 22.34 years (SD = 11.43). Non-verbal intelligence was estimated using the RCPM (Raven’s Colored Progressive Matrices (RCPM) score < 16; Carlesimo et al., 1996), a non-verbal test that is considered a measure of fluid intelligence (Irwing & Lynn, 2005; Snow & Lohman, 1989). On average, the SSD group displayed a non-verbal intelligence score that was within normal limits (Table 1). Thirty Healthy Control (HC) participants, matched by age-class (18–30, 31–40, 41–50, and 51–65), gender, and education, were recruited among the hospital staff and the general population. Exclusion criteria for the control group were the same as those for the groups of participants with SSD, plus any documented psychiatric disorders or being first-degree relative of a patient with a diagnosis of SSD. All participants were native speakers of Italian. Socio-demographic and clinical data of the study sample are reported in Table 1.

**Table 1:** Socio-demographic and clinical data of participants at recruitment

Variables	SSD	HC	t	*P* value
	*N* = 30	*N* = 30		
Age, mean years (SD)	47.73 (9.8)	47.7 (10.58)	− 0.01	.99
Education, mean years (SD)	10.32 (3.73)	11.76 (3.42)	1.67	.10
Gender	20 M + 10 F	20 M + 10 F		
SSD diagnosis				
Schizophreniform disorder	1 (3%)			
Schizophrenia	18 (60%)			
Schizoaffective disorder	7 (23%)			
Other SSD	4 (14%)			
Illness duration, mean years (SD)^a^	22.34 (11.43)			
Antipsychotic medications				
First generation only	22			
Second generation only	2			
Both	6			
Symptomatology (BPRS-E)	43.7 (10.25)			
Nonverbal intelligence (RCPM)	27.73 (5.84)			

BPRS-E, Brief Psychiatric Rating Scale-Expanded; RCPM, Raven’s colored progressive matrices; HC, healthy controls; SSD, schizophrenia spectrum disorder; SD, standard deviation

^a^Data available for n = 29 SSD participants

After having presented the study, written informed consent to participate was obtained from all participants. The study was approved by the IRCCS Ethical Committee (Opinion 61/2017) and followed the principles of the Helsinki Declaration.

### Stimuli

A set of 112 sentences was created ad hoc for the present study. The list of Italian verbs was partially derived from Vernice and Sorace (2018), although the final set of sentences was substantially modified to fit the needs of the present study. Items were validated on a sample (N = 20) of healthy participants (not included in the final study sample), through a rating study: subjects were asked to rate each sentence on a 5-point scale, where 1 was “totally unacceptable” and 5 was “totally acceptable”. Sentences that were rated differently from the intended categorization were either removed or replaced. In half (N = 56) of the stimuli, the grammatical subject of the sentence was the “Agent” of the verb (Table 2, conditions 1a and 1b); in the other half (N = 56), the subject was the “Theme” of an unaccusative verb (Table 2, conditions 2a and 2b). In both cases, subjects were always in preverbal position (i.e., they appeared before the verb). Half of the sentences (N = 56) were semantically acceptable (Table 2, conditions 1a and 2a), and the other half (N = 56) not acceptable (Table 2, conditions 1b and 2b). The semantic anomaly was an incongruent animacy (i.e., the state of being alive and animate) feature of the grammatical subject (Table 2 reports examples of the stimuli). Semantic violations always affected the grammatical subject in the pre-verbal position to avoid a possible confounding effect related to word position within the sentence. The same experimental list was administered to all participants, with sentences appearing in random order. The complete set of stimuli is provided in the Supplementary Materials.

**Table 2:** Experimental stimuli

Condition	Example	N. of stimuli
1a. Agent—correct	Ogni sera Bianca telefona al nipotino *(Every evening Bianca calls her nephew)*	28
1b. Agent—with violation	* Ogni sera il sonno telefona al nipotino *(Every evening the sleep calls its nephew)*	28
2a. Theme—correct	A volte il bambino cade dalle scale *(Sometimes the child falls from the stairs)*	28
2b. Theme—with violation	* A volte il sole cade dalle scale *(Sometimes the sun falls from the stairs)*	28
3a. Filler—Subject-verb agreement	Nel bosco le volpi *scappa dal cacciatore (*In the woods the foxes runs away from the hunter*)	8
3b. Filler—Determiner-NP	Nel mare *le pesci nuotano liberi (*In the sea the*_*FemPlur*_* fishes*_*MascPlur*_* swim free*)	8
3c.Filler—Verb-clitic	Il cane insegue il gatto e *la rincorre fin sotto il tavolo (*The dog chases the cat*_*MascSing*_* and runs after her*_*FemSing*_* under the table*)	8

Conditions, examples, and numbers of experimental stimuli. Target verbs are underlined. A literal translation is reported in brackets

Given that frequent words are processed faster than less frequent words (Brysbaert et al., 2011) and that long words slow down reading times (Rayner et al., 2011) frequency and length of target words (verbs) were matched across conditions (Table S3, Supplementary Materials). Frequencies of target words were taken from Subtlex-IT (available at http://crr.ugent.be/subtlex-it/).

Twenty-four sentences containing morphological violations were added as fillers and randomly presented to participants along with target sentences. The subset of morphologically violated sentences was structured as follows: eight sentences contained a violation on the subject-verb number agreement (i.e., singular subject with a plural verb, and vice-versa; e.g. “*Nel bosco le volpi scappa dal cacciatore”/ “*In the woods the foxes runs away from the hunter”), eight sentences contained a violation on the determiner-noun agreement (e.g. “*Nel mare le pesci nuotano liberi”/ “*In the sea the_FemPlur_ fishes_MascPlur_ swim free”), and eight sentences contained a violation of the agreement between a clitic pronoun (i.e., unstressed, phonologically bound pronoun) and its NP-antecedent (e.g. “*Il cane insegue il gatto e la rincorre fin sotto il tavolo”/ “*The dog chases the cat_MascSing_ and runs after her_FemSing_ under the table”). The complete set of items is provided in the Supplementary Materials.

### Eye-Tracking Procedure

An Eye-link 1000 Plus® was employed to collect data on eye-movement (1000 Hz with a single 35 mm lens), calibrated using a three-point grid before each acquisition with chin-rest support. The Eye-link 1000 Plus® is a corneal reflection system that assesses changes in gaze position by measuring both the reflection of an infrared illumination on the cornea and the pupil size by means of a video camera sensitive to the light in the infrared spectrum. For this study, we used a desktop camera and a chin-rest support to stabilize participants’ head position. The left eye was recorded. The experimental stimuli were presented on a Display PC via Experiment Builder®, an object-oriented programming suite specifically designed to administer stimuli while recording eye movements (for details on eye-tracking procedures and measures, see Duchowski, 2017; Rayner, 1998; and Sharma et al., 2021). Sentence presentation was preceded by a fixation point and triggered manually by the experimenter. Participants were asked to silently read each sentence and then evaluate their acceptability by pressing either a green (acceptable) or red (not acceptable) key on the keyboard. Each sentence remained on the screen until one of the keys was pressed.

### Data Analysis

We used three indices of reading process as dependent variables: Gaze Duration (GD), probability of regression (go-back movements), and Total Fixation Duration (TFD). First Fixation Duration (FFD) and accuracy of responses were also collected: analyses for these two variables are reported in the Supplementary Materials. GD is defined as the sum of all fixations made on the target word before the eyes leave the region. This metric is considered a measure of lexical access (Rayner et al., 2011), which, in the case of verbs, includes processing of the argument structure (Bock & Levelt, 1994). Probability of regression is, in our study, the probability to register a go-back movement starting from the verb and moving back towards the subject. Regression movements are associated with integration difficulties (Reichle et al., 1998), in our case, between the verb and its pre-verbal grammatical subject. TFD is the cumulative duration in milliseconds of all fixations on the target word, including any regression; this measure is generally considered to provide insight into any possible processing difficulties associated with the integration of the word meaning within the sentence (Rayner et al., 2006).

Differences in probability of regression in the two groups were tested with a 2-sample test for equality of proportions with continuity correction. Separate statistical models were run on each index of reading process as dependent variable, with (thematic) *Role*, *Condition*, and *Group* as categorical independent variables. Data on fixations (GD and TFD) were logarithmically transformed to reduce the skewness of distributions, thus obtaining a Gaussian-like distribution (Baayen, 2008). As each independent variable had two levels (Agent *vs* Theme, Correct *vs* With Violation, and Participants with SSD *vs* HCs, respectively), our study had a mixed 2 × 2 × 2 factorial design. Linear mixed-effects regression analyses (Baayen et al., 2008) and generalized linear mixed-effects models (Baayen et al., 2008) were conducted. Two-way and three-way interactions were included in the model. Random intercepts for items and participants were also included. All models were refitted after having identified and excluded atypical outliers using a 2.5 SD criterion over the model standardized residuals (in order to exclude substantial impact of outlying datapoints, according to the “model criticism” approach proposed by Baayen et al., 2008). Statistics of the refitted models are reported. Logistic mixed-effects regression analyses (Jaeger, 2008) were run on regression data, with participants and items as crossed random effects (Baayen et al., 2008). Pairwise post-hoc comparisons using R’s multcomp package (Simultaneous Test for General Linear Hypothesis—glht) were run in the case of significant three- or two-way interactions (Howell & Lacroix, 2012). Alpha level was set at 0.05. All the analyses were performed in R, v.3.6 (R CoreTeam, 2019).

## Results

Data of interest were fixation duration on verbs (i.e., GD and TFD) and backwards eye-movement from the verb toward the grammatical subject (i.e., the probability of re-fixating the grammatical subject after having read the verb). The total number of valid data-points was 6,105 out of 6,480 (94.21%), equally distributed between groups (HC = 3,092; SSD = 3,013). Similarly, data were equally distributed across conditions in both groups (around one-fourth of the total number of valid fixations for each condition and each group). Table 3 summarizes the mean duration (in milliseconds) and standard error of the mean GD and TFD on target verbs, as well as the probability of regression from target verbs, per conditions and in the two groups. Table 4 shows the estimated fixed and random parameters of the model for GD, probability of regression, and TFD, together with significance tests.

**Table 3:** Gaze duration, total fixation duration, and probability of regression: descriptive statistics

	SSD				HC			
	Agent		Theme		Agent		Theme	
	M	SEM	M	SEM	M	SEM	M	SEM
*GD*								
Correct	519.02	16.11	484.17	12.40	362.81	7.12	382.22	7.88
With violation	545.40	16.08	527.72	13.05	420.78	9.35	421.18	9.96
*TFD*								
Correct	1197.90	48.01	1118.41	42.85	602.62	14.84	597.64	14.38
With violation	1379.39	52.42	1470.16	54.04	620.27	14.49	635.11	16.35
*Probability of regression*								
Correct	80%		76%		71%		73%	
With violation	84%		84%		75%		73%	

Mean durations and standard error of the mean (in milliseconds) of GD and TFD on target verbs, and probability of regression from target verbs backwards, per conditions, in the two groups

GD, gaze duration; HC, healthy controls; M, mean; SEM, standard error of the mean; SSD, schizophrenia spectrum disorder; TFD, total fixation duration

**Table 4:** Estimated fixed and random parameters for GD, probability of eye-movement regression from the verb, and TFD

Random effects	GD		Probability of regression		TFD	
	Variance	SD	Variance	SD	Variance	SD
Subject (intercept)	.0779	.2790	1.3583	1.1654	.1906	.4366
Item (intercept)	.0201	.1420	.3298	.5743	.0318	.1783
Residual	.1412	.3758			.2541	.5041

Fixed effects	Estimate	SE	df	t-value	*p*	Estimate	SD	Z value	*p*	Estimate	SD	df	t-value	*p*
Intercept	5.79	.06	93.42	97.86	< .001	1.12	.26	4.39	< .001	6.21	.09	82.43	70.23	< .001
Condition (violated)	.14	.02	5829.22	7.16	< .001	.30	.13	2.38	.017	.07	.03	5898.00	2.85	.004
Role (Theme)	.06	.04	72.20	1.31	.194	.16	.20	.80	.426	− .01	.05	80.55	− .11	.911
Group (SSD)	.25	.07	64.47	3.36	.001	.68	.33	2.05	.041	.48	.12	62.68	4.13	< .001
Condition * Role	− .09	.03	5839.91	− 3.23	.001	− .32	.18	− 1.79	.074	− .02	.04	5910.00	− .59	.558
Condition * Group	− .08	.03	5807.75	− 3.06	.002	.11	.19	.59	.558	.12	.04	5876.00	3.22	.001
Role * Group	− .07	.03	5807.72	− 2.70	.007	− .43	.19	− 2.30	.022	− .04	.04	5876.00	− .99	.323
Condition * Role * Group	.1	.04	5807.64	2.58	.010	.58	.27	2.13	.033	.06	.05	5876.00	1.23	.219

df, degrees of freedom; SD, standard deviation

### Gaze Duration

**Fig. 1:**
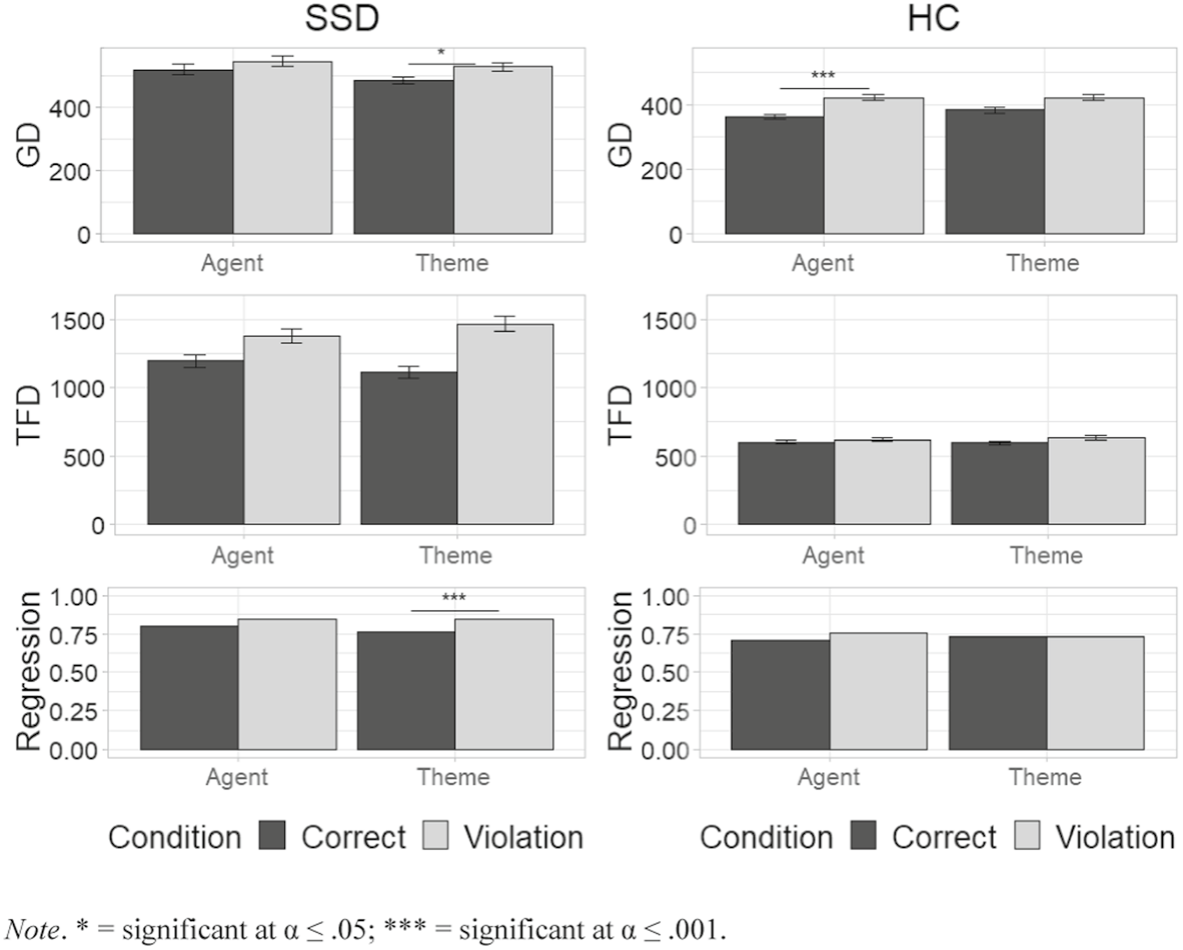
Mean GD, FTD and probability of regression by group, role, and condition (bars indicate the standard error of the mean). *Note* *significant at α ≤ .05; ***significant at α ≤ .001

A significant three-level interaction (t = 2.58, *p* = 0.010) on GD indicates that the effects of *Condition* and *Role* interacted differently in the two experimental groups at this stage of the sentence processing. Results (Fig. 1) show an opposite pattern in the two groups when reading verbs carrying a semantic violation of the Agent subject (e.g., “*Ogni sera il sonno telefona al nipotino”/ “*Every evening the sleep calls its nephew”) *vis-à-vis* verbs carrying a semantic violation of the Theme subject (e.g., “*A volte il sole cade dalle scale”/ “*Sometimes the sun falls from the stairs”).

Post-hoc comparisons showed that HCs displayed significantly longer GDs when reading verbs that carry a semantic violation on their Agent subjects, *vis-à-vis* control sentences (z = − 7.16, *p* < 0.001). However, HCs did not show such lengthening when reading verbs with a violation in which the incongruent subject was a Theme, *vis-à-vis* control sentences (z = − 2.54, *p* = 0.11). On the contrary, GD in participants with SSD was significantly longer in case of verbs carrying a violation of the Theme subject *vis-à-vis* control sentences (z = − 3.306, *p* = 0.012) and, conversely, GD in this group was not significantly longer when reading verbs carrying a violation of the Agent subject, *vis-à-vis* control sentences (*p* = 0.07).

### Probability of Regressions

Probability of regression was rather high in both groups (> 70%), with SSDs participants showing an overall higher (M = 81.45%) probability than HCs (M = 73.06%; *p* < 0.001) of regression from the verb in all conditions (Fig. 1). We thus further explored the effects of *Condition* and *Role* in the two experimental groups on the probability to re-fix the grammatical subject after having read the target verb.

A logistic mixed-effect model having the probability of regression as dependent variable (yes–no) and *Condition*, *Role*, and *Group* as predictors was run. A significant three-level interaction (z = 2.13, *p* = 0.033) between the fixed effects of *Condition*, *Role*, and *Group* was found, meaning that, similarly to what was observed for GD, the effect of the independent variables *Role* and *Condition* acted differently on the probability of regression for the two experimental groups. Post-hoc analyses indicated that the interaction between *Condition* and *Role* did not affect the probability of regression in HC. This group did not show a higher probability of re-reading the pre-verbal subject in sentences with a semantic violation than in control sentences, neither in the case of Agent subjects (z = − 2.38, *p* = 0.19) nor in the case of Theme subjects (z = 0.16, *p* = 0.99). In the SSD group, the interaction between *Condition* and *Role* did not affect the probability of moving back from the verb to the subject in sentences with an Agent subject (z = − 2.79, *p* = 0.07). However, it significantly affected the probability of regression movements in sentences with a Theme subject (z = − 4.57, *p* < 0.001).

### Total Fixation Duration

To explore the cognitive mechanisms underlying the late stage of sentence processing, we entered our variables of interests (*Condition*, *Role*, and *Group*) as categorical predictors in a mixed-effect model having TFD as a continuous dependent variable.

**Fig. 2:**
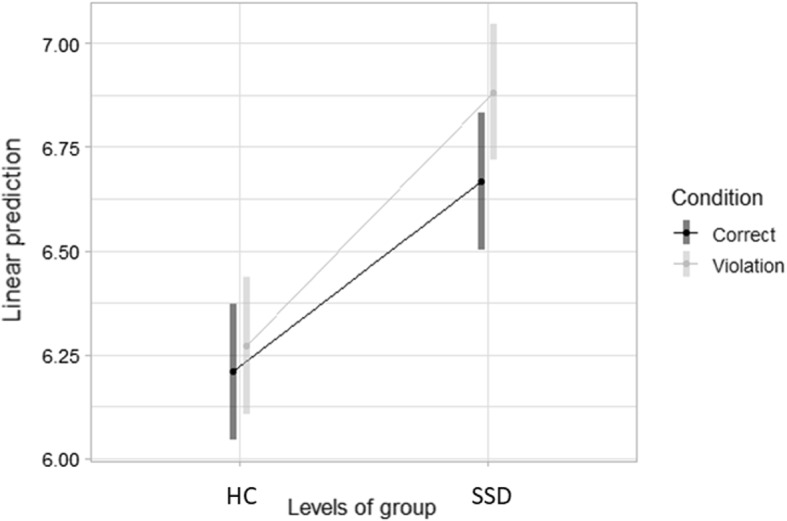
Two-way interaction plot on TFD by Group and Condition. *Note* *HC* healthy controls, *SSD* schizophrenia spectrum disorders

No three-level interaction between the three fixed predictors was found, indicating that the effects of *Condition* and *Role* did not interact differently in the two groups on measures of later-stage processes. A two-level interaction between *Condition* and *Group* was found (t = 3.22, *p* < 0.001), indicating that the presence of a semantic anomaly on the subject (irrespective of its thematic role) might exert a different effect in the two groups (Fig. 1). Post-hoc analyses (Fig. 2) indicated that the interaction between *Condition* and *Group* significantly affected TFD both in HC and in SSD participants (z = − 3.43, *p* < 0.001 and z = − 11.33, *p* < 0.001, respectively). Estimated marginal means by Group showed an average increase in reading times for HC (− 0.06 ms) and SSD participants (− 0.21 ms).

## Discussion

The present study aimed to investigate the integrity of the cognitive representation of the thematic grids of verbs in people with SSD. Based on studies that identified an impaired verbal (action) fluency (Badcock et al., 2011; Kambanaros et al., 2010; Marvel et al., 2004; Smirnova et al., 2017; Woods et al., 2007) and an impaired sense of Self (Haug et al., 2012; Henriksen & Noordgard, 2014; Postmes et al., 2014; Nordgaard et al., 2018) possibly related with the linguistic knowledge of the “Agent” thematic role, we expected higher tolerability to a semantic violation on the Agent in people with SSD than in the healthy population. Differently from HC participants, we expected people with SSD to be less impacted by animacy violations on the Agent subject, indicating the lack of an (implicit) anomaly detection process, along with the suboptimal integration abilities already observed in this clinical population (Kuperberg et al., 2006a; Whitford et al., 2017).

To do so, we recruited a cohort of participants with and without SSD, matched for age, gender, and education, and we collected eye-movement measures during a silent reading task. In half of the stimuli, grammatical subjects (either an Agent or a Theme) carried a violation of the animacy aspect, creating an unacceptable Thematic Grid for the sentence verb (e.g., “*Ogni sera il sonno telefona al nipotino”/ “*Every night the sleep calls its nephew”—in this specific example, the thematic grid of the verb “telefonare”/ “*to call*” requires an animate Agent). A “lexicalist” approach to language processing, posing that the verb thematic grid is part of its lemma representation (Bock & Levelt, 1994), would predict an effect on the implicit measures of lexical access to the predicate components, i.e., gaze duration on the target verb. Moreover, based on previous evidence of syntactic-semantic integration anomalies (Kuperberg et al., 2006a) pointing to a suboptimal use of linguistic context to process and produce speech as well as to difficulties in high-order integration processes when reading (Roberts et al., 2013; Fernández et al., 2016), additional general-level effects could be expected in both measures of local and sentence integration, i.e., regression movements and TFD, respectively.

As for measures of lexical access (GD), our results indicate that the effects of our variables of interests, *Condition* and *Role,* interact differently in the two groups. Sentences carrying a violation of the animacy feature of the Agent appear to be more tolerated by people with SSD than by HCs, as this combination of variables did not affect GDs on the target verb. In other words, people with SSDs did not increase the processing time for the verb, visually processing the anomalous stimulus as if it were correct. Conversely, the HC group was more sensitive to semantic violations when the affected subject was an Agent, compared to violations on the verb Theme. This finding suggests a different saliency of semantic violations for the two thematic roles in the two experimental groups, and particularly that animacy violations on the Agent grammatical subject are better tolerated in the SSD population. We interpret these results as an indication of a disrupted lexical representation of the verb thematic grid in the SSD population, which seem to hinder the ability of people with SSD to evaluate the appropriateness of the Agent animacy features.

These results further support and extend studies on SSD that employed word-monitoring tasks (Kuperberg et al., 1998), which highlighted an insensitivity to linguistic violations by participants with schizophrenia, as well as analyses on electrophysiological responses (Kuperberg et al., 2006b) on critical verbs, suggestive of abnormalities in combining semantic and syntactic information online to build up propositional meaning. Moreover, our results support previous results on the presence of oculomotor markers of reading difficulties in SSD (such as increased GD and TFD, and increased probability of regression compared to HCFernández et al., 2016; Roberts et al., 2013; Whitford et al., 2013) and complement them by showing the impact of different thematic roles. According to Levelt’s theory of lexical access (Bock & Levelt, 1994; Levelt et al., 1999), a speaker selects a lemma (i.e., the cognitive representation of a word) from the mental lexicon and, in doing so, the active lexical concept spreads some of its activation to its lemma node; in the case of verbs, these are specified by the verb argument structure and its thematic grid. Upon the selection of a lemma, its syntax becomes available to create the appropriate syntactic environment for further grammatical encoding. We suggest that, in people with SSD, this top-down linguistic processing could be linked to the disrupted sense of agency observed in this clinical population. Albeit a univocal characterization of the Agent thematic role of a verb cannot be given, the group of HCs appear to share an overall agreement that certain subjects (animate nouns) are likely to “act”, whereas others (inanimate nouns) are not. Linguistically speaking, the Thematic Grid Knowledge appears coherently shared in HCs, and, as a result, the conceptual representation of agency is consistently translated into lexicalized items. Such shared representations in the lexical store are then used to process the subject-verb relationship during reading. On the contrary, in people with SSD such knowledge appears to be somehow impaired, allowing for anomalous subjects to fill the role of Agent. We suggest that the disrupted representation of agency as observed in SSD might be implied in the integration processes between subjects and verbs. Although our results cannot allow any conclusions on the directionality of this effect, a primary linguistic misrepresentation of the thematic grid knowledge may affect the ability to properly attribute agency (Crow, 2008; Hinzen & Rossello, 2015). However, the reverse direction of the effect is also possible, whereby a primary bottom-up misrepresentation of one own’s action may cause an impaired lexical representation of the thematic grid knowledge (for first-person actions) and possibly extending to actions performed by others (Lindner et al., 2005; Synofzik et al., 2010).

As for the probability of regression from the verb, our group of participants with SSD showed a higher probability of regression in all conditions than the group of healthy controls; in this latter group, in fact, a violation did not affect the probability of re-reading the pre-verbal grammatical subject, irrespective of its Thematic Role. This result is coherent with previous literature and indicates a significant effect of integration load on neurophysiological measures of reading in people with SSD (Fernández et al., 2016; Kuperberg et al., 2006b; Roberts et al., 2013; Whitford et al., 2013). To the best of our knowledge, this is the first study to extend such observation to eye-tracking measures in reading connected texts with semantic anomalies. In normal conditions, the identification of a semantic anomaly would prompt the reader to go back to the element that could presumably help to disambiguate the conceptual conflict (in this case, the pre-verbal subject). However, we found that participants with SSD were likely to re-read the subject of a sentence containing a semantic violation on the Theme grammatical subject, but, conversely, the presence of the same type of semantic violation did not affect the probability of regression when the element carrying the semantic violation was an Agent subject. This means that a semantic violation on the pre-verbal Theme as grammatical subject was salient for this group, since it significantly increases the probability of a regression movement, unlike what was observed for a violation of the Agent. Again, this result speaks for a lack of sensitivity, at the local level, to violations on the Agent in the SSD group, as observed for GD. As go-back movements during reading have been linked to integration difficulties (Reichle et al., 1998), we interpret this result as indicative of an impaired ability to identify online semantic anomalies involving an Agent as grammatical subject, leading to a reduced sensitivity to violations on this linguistic structure. These results are indeed in line with our findings on GD measures and demonstrate both the psychological reality of Thematic Roles and a disrupted lexical representation of verb Thematic Grid in SSD individuals.

At the end-of-sentence stage, we observed an interaction between the effects of the main variables *Group* and *Condition* on TFD, a measure of cognitive processing previously linked to integration difficulties of the word meaning within the sentence (Rayner et al., 2006). Specifically, post-hoc comparisons highlighted significantly longer TFDs when reading sentences with a violation for both HCs and participants with SSD. This finding may reflect late integration and re-analysis processes of structural and lexical-semantic information at the sentence level in conjunction with a semantic violation. A greater magnitude of the effect was observed for the SSD sample, in line with the model proposed by Kuperberg et al. (2010) about a specific integration difficulty between syntactic and semantic information in sentence comprehension in people with SSD. In other words, a semantic anomaly on the verb thematic grid determined an increasing length of TFD, with participants with SSD showing a greater effect than HCs.

One may argue that these results are linked, at least in part, to the fact that constructs implying syntactic movement (unaccusative verbs with pre-verbal Theme as grammatical subject) pose an additional cognitive load to the reader. The task employed in the present study requires the subject to store and integrate syntactic (pre-verbal movement of the subject), morphological (argument structure), and semantic information (animacy of the thematic roles) in the working memory store. In such a frame, the impaired working memory and executive functions in people with SSD (Gold et al., 2002) may have negatively affected the performance of these individuals. However, in this case, we should have found, irrespective of violations, longer GDs for the allegedly cognitively heaviest sentences (those with a Theme subject), compared to sentences with an Agent subject. This was not the case, ruling out such alternative, syntax-centered, explanation. From a more pragmatically centered point of view, one may claim that, when reading sentences with unaccusative verbs depicting changes of states, it is more difficult to exclude the implausibility of inanimate grammatical subjects. In other words, it could be that sentences such as “The sun falls from the sky” are more acceptable than sentences such as “The sleep gives a call”. However, if this were the case, we would have found the same effect for both groups, i.e., the semantic anomaly on the Theme subject would have been difficult to recognize both by HPs and participants with SSD, leading to significantly longer GDs for sentences with an anomalous Theme subject in both groups. On the contrary, we observed a double dissociation for group and thematic role: while HPs took significantly longer to process an anomalous Agent as such, they could easily spot a correct Agent. Previous studies found that Agents provide more information about event structure than do Themes, and, therefore, facilitate event processing in healthy participants (Cohn et al., 2017). The reverse pattern emerged in our experimental group: for participants with SSD, it was significantly easier to process an anomalous Theme than a correct one, but we observed no difference when this group of participants processed an anomalous Agent compared to an appropriate one.

It must be noted that a fundamental aspect of the present study is lexical access. This process entails linking the written symbol (the word) and its corresponding representation from long-term memory. One factor associated with prolonged lexical access time is the presentation of a word in an incongruous context. From a neurophysiological perspective, the Evoked-Related Potential (ERP) that best mirrors this process is the N400. Studies investigating the effect of semantic violations through selection restrictions (i.e., anomalies derived from failing to comply with requirements posed by the verb on nouns that can take part to a certain action) consistently elicited a monophasic increase in N400 amplitude (De Vincenzi et al., 2003; Osterhout & Mobley, 1995; Osterhout & Nicol, 1999), a negative peak in ERP that appears around 400 ms after the onset of the anomalous stimulus. From a theoretical, as well as time-related, point of view, it appears likely that our GD measure reflects the N400 component found in ERP studies. In this frame, GDs seem to mirror the semantic integration processes of words with its preceding context and indicate that such integration is strongly influenced by the congruency of grammatical subjects with the thematic role assigned by the verb. Our findings support the assumption that syntactic-semantic incoherence is processed at the local level, with animacy playing a role in influencing the computation of thematic relationships between a verb and its argument(s) during online sentence comprehension in healthy participants (Kuperberg et al., 2007) but less so in people with SSD. Later stages of linguistic processing, when structural and lexical-semantic representations of the sentence are integrated, have been identified to be related to the P600 component (Friederici, 2002), a positive ERP component that appears about 600 ms after the onset of the critical stimulus. If there are problems with the integration of the syntactic and semantic representation of a sentence, the P600 indicates the process of sentence re-adjustment or re-analysis (when possible). Similarly, in our study, such a late stage of sentence processing appears to be mirrored in the TFD measure and appears to be active both in HCs and in participants with SSD.

Our results are consistent with previous modular models of language comprehension in healthy subjects (Frazier & Rayner, 1982, 1987) as well as in people with SSD (Kuperberg et al., 2006a; Ruchsow et al., 2003). Along these lines, we propose a possible model of anomalous sentence processing in SSD, developing, temporally speaking, along two stages:

1. Early access to an impaired lexical representation of the verb (indexed by GD). As soon as the sentence main verb is accessed, syntactic and semantic information stored in the Thematic Grid of the verb argument structure is retrieved from the mental lexicon. In SSD participants, the disrupted lexical representation of the verb thematic grid, linked to an altered mental representation of agency, leads to a different saliency of semantic violations on the animacy trait of an Agent-subject, and, in turn, an increased tolerance of this kind of violations. It follows that no regression movement from the verb to the pre-verbal grammatical subject, needed to disambiguate the semantic anomaly at the local level, is triggered. In other words, at this first stage, participants with SSD overlook the semantic anomaly in the Agent pre-verbal subject, while displaying a distinguished sensitivity for a violation on the Theme.
2. A later resolution of the semantic anomaly by integrating syntactic and semantic information at the sentence level (mirrored by TFD). At the end of the sentence, both HCs and participants with SSD show an effect of the semantic violation, with longer TFD when reading sentences with a violation of the subject (regardless of its Thematic Role). However, the magnitude of the effect seems to be particularly evident in the SSD group. This result speaks for a general-level integration difficulty in participants with SSD when facing a semantic violation.

The present study bears at least two major limitations that are worth mentioning. Our group of participants with SSD has a mean age of 48 year and a mean history of illness of about 22 years. This means that, at the time of evaluation, this group had been in treatment with at least one anti-psychotic medication for nearly all their adult life. Albeit results from previous studies investigating eye movements during connected reading have ruled out any correlation between antipsychotic medications and ocular motor parameters (Roberts et al., 2013; Fernández et al., 2016; Whitford et al., 2013), our results were not controlled for the possible effect of medication on the performance to the task. Moreover, at time of recruitment, the majority (63%) of participants in the SSD group were hospitalized. This means that our SSD group represents a subgroup of the SSD population, selected for severity of the disorder. For this reason, the present results are not necessarily generalizable to the entire SSD population.

## Conclusions

This study shows the specific contribution of a disrupted lexical representation involving the thematic grid structure to the reading performance in individuals with SSD. Our results support the hypothesis of an impaired lexical representation of verbs requiring an animated Agent as the grammatical subject in this clinical population. A disrupted representation, which we link to the presence of a “disordered self,” affects the implicit processing of sentences, as captured by GDs and probability of regression in reading.

This study identifies an overall difficulty in processing the concept of “agentivity” in people with SSD, which might help explaining some clinical phenomena such as delusions of persecution (e.g., being intentionally followed by the wind). Moreover, the results of the present study might contribute to the understanding of the so-called “word salad” occasionally found in people with schizophrenia, i.e., incoherent speech made of bizarre and sometimes nonsensical word associations. In turn, a refined formalization of linguistic deficits in SSD might contribute to the development of novel psychosocial treatments. Indeed, cognitive rehabilitation has been a component of some psychosocial treatment packages for SSD (Vita et al., 2021). However, although speech disturbances in SSD have been linked to a lower quality of life (Tan et al., 2014), the attention paid to overt linguistic training is limited in current rehabilitation protocols. A possible complementary approach could be derived from rehabilitation programs developed for patients with aphasia who display similar language deficits. For example, short-term intensive therapy targeting argument structure, aimed at generalization and reinforcement of simple sentence structures (Bazzini et al., 2012), suggests that direct and indirect training of deep linguistic structures can translate into long-lasting effects on general communication abilities.

## Supplementary Information

Below is the link to the electronic supplementary material.

Supplementary file1 (DOCX 1696 kb)

## Data Availability

The data that support the findings of this study are available upon request and with the permission from the IRCCS Istituto Centro San Giovanni di Dio Fatebenefratelli. Data are not publicly available due to privacy restrictions.

## Footnotes

1 Following the standard linguistic usage, an * indicates an anomalous syntactic sentence or a lexical mistake.
