# Supplementary material for: Agency of Subjects and Eye Movements in Schizophrenia Spectrum Disorders
Source: J Psycholinguist Res. 2022 Jul 16;51(6):1371–91. doi: 10.1007/s10936-022-09903-6 (PMC9646601; doi:10.1007/s10936-022-09903-6)
Supplement: Supplementary file 1 — Supplementary file1 (DOCX 1696 kb) [file 10936_2022_9903_MOESM1_ESM.docx]

**Supplementary Materials**

**Table S.1**

*Target stimuli.* Items were validated through a rating study: twenty healthy participants were asked to rate each sentence on a 5-point scale, where 1 indicated a “totally unacceptable” and 5 a “totally acceptable” sentence. Sentences that were rated differently from the expected categorization were replaced and the new items were reassessed. The expected position of the animated Agent in canonical transitive sentences is the first one (Paczynski & Kuperberg, 2011)^[[1]](#footnote-1)^, while post-verbal slots are generally reserved to other arguments. In sentences with unaccusative verbs, word order is the result of a syntactic movement that takes the direct post-verbal object and moves it to the position of the pre-verbal grammatical subject (Burzio, 1986; Perlmutter, 1978)^[[2]](#footnote-2)^. A relevant feature of unaccusative verbs relies on their semantic underpinnings, in the sense that unaccusative verbs require a non-agentive argument (Dowty, 1991; Van Valin, 1997)^[[3]](#footnote-3)^.

| **AGENT - correct** | **AGENT – with violation** | **THEME - correct** | **THEME – with violation** |
| --- | --- | --- | --- |
| In estate la luce brilla attraverso le ante | Nel cantiere l’architetto solleva la cisterna | Al mattino le stelle tramontano dietro alla collina | In paese le fontane cambiano a ogni generazione |
| Alla luce i diamanti luccicano nell’astuccio | In inverno la neve riposa nella grotta | In paese gli abitanti cambiano a ogni generazione | Davanti al camino la zia rimane calda |
| Ogni sera Bianca telefona al nipotino | Ogni sera il sonno telefona al nipotino | A volte il bambino cade dalle scale | A volte il sole cade dalle scale |
| Sulla roccia il leone ruggisce con fierezza | Sulla roccia l'arbusto ruggisce con fierezza | In estate il maestro parte per la montagna | In estate la tana parte per la montagna |
| Alla festa gli uomini ridono di gusto | Alla festa i bicchieri ridono di gusto | All’improvviso il ragazzo sviene per la paura | All’improvviso l’oro sviene per la paura |
| In cucina i gatti dormono al sole | In cucina le bucce dormono al sole | Tutti i giovedì il professore arriva in ritardo | Tutti i giovedì il cielo arriva in ritardo |
| In estate i calciatori giocano all'aperto | In estate le panchine giocano all'aperto | Ogni giorno molti soldati muoiono nel mondo | Ogni giorno molte scarpe muoiono nel mondo |
| Nella favola la regina abdica al trono | Nella favola la colonna abdica al trono | A mezzogiorno i pensionati vanno in banca | A mezzogiorno i negozi vanno in banca |
| A pranzo il papà sbuccia l’arancia | A pranzo il porto sbuccia l’arancia | Grazie alla pedana l’elefantino scende dal camion | Grazie alla pedana il cruscotto scende dal camion |
| Sul marciapiede il ciclista gonfia la ruota | Sul marciapiede il parchimetro gonfia la ruota | Alle sei gli operai tornano a casa | Alle sei i ristoranti tornano a casa |
| La mattina i postini consegnano le lettere | La mattina i cereali consegnano le lettere | Ogni giorno molti neonati nascono in ospedale | Ogni giorno molti treni nascono in ospedale |
| Nel fine settimana i cuochi cucinano le torte | Nel fine settimana i parchi cucinano le torte | A mezzanotte i fuochi scoppiano nel cielo | A mezzanotte gli innamorati scoppiano nel cielo |
| Tutti i giorni la mamma legge il giornale | Tutti i giorni il muro legge il giornale | All'aeroporto gli aerei decollano a ogni ora | In primavera la gallina sboccia nel campo |
| In autostrada il camionista guida con prudenza | In autostrada l'asfalto guida con prudenza | In primavera il fiore sboccia nel campo | Questa settimana l'avvocato tramonta al mattino |
| Ogni sera sua moglie regola la sveglia | Ogni sera la notizia regola la sveglia | Con la convalescenza i malati guariscono dalla malattia | Con la convalescenza gli ospedali guariscono dalla malattia |
| D’estate le onde bagnano la riva del mare | D’estate le tasse bagnano la riva del mare | Dopo le feste molte persone ingrassano di qualche chilo | Dopo le feste molti piatti ingrassano di qualche chilo |
| A pranzo gli atleti mangiano i maccheroni | A pranzo i gomiti mangiano i maccheroni | All’improvviso le rovine franano rumorosamente | All’improvviso le lavoratrici franano rumorosamente |
| Di giorno i cani abbaiano alle automobili | Di giorno le rose abbaiano alle automobili | Sul balcone le fragole spuntano nei vasi | Sul balcone le insegnanti spuntano nei vasi |
| La sera i pesci abboccano con facilità | La sera i dischi abboccano con facilità | Sul monte gli alberi fioriscono a primavera | Sul monte gli studenti fioriscono a primavera |
| Nel castello la spada scintilla accanto al camino | Nel castello lo stalliere scintilla accanto al camino | La sera i fulmini lampeggiano sulla montagna | La sera i cervi lampeggiano sulla montagna |
| A mezzogiorno le campane risuonano nella valle | A mezzogiorno le ballerine risuonano nella valle | Col caldo l'acqua evapora dalla pozzanghera | Col caldo il dottore evapora dalla pozzanghera |
| All’occorrenza i pompieri corrono in aiuto | Quando occorre i papaveri corrono in aiuto | Dopo la sconfitta il nemico fugge sulle montagne | Col maltempo il caffè rimane al porto |
| Nel cantiere la gru solleva la cisterna | Nel cielo il gufo brilla a primavera | Col maltempo la nave rimane al porto | Dopo un terremoto le macerie intervengono con tempestività |
| In ufficio i telefoni squillano senza sosta | In ufficio le mosche squillano senza sosta | Dopo un terremoto i pompieri intervengono con tempestività | Alle quattro i telefoni escono dall'ufficio |
| A Natale il nonno regala qualche caramella | Alla luce gli attori luccicano nell’astuccio | Alle quattro gli impiegati escono dall'ufficio | Il mercoledì il cane costa di meno |
| Sulla montagna il vento soffia indisturbato | Sulla montagna il falco soffia indisturbato | Il mercoledì il cinema costa di meno | In Niger le madri scarseggiano durante la carestia |
| Sul banco la cartella contiene i libri | Durante l’intervista il microfono regala una copia del film | In Niger le risorse scarseggiano durante la siccità | Grazie alle dighe i manichini sopravvivono all'inverno |
| In città i grattacieli luccicano sotto il sole | Sotto al tavolo la mattonella solleva una briciola | Grazie alle dighe i castori sopravvivono all'inverno | In estate il macellaio scade dopo pochi giorni |
| Nel cielo il sole brilla a primavera | D'estate il sasso riposa sotto l'albero | In America i mandati presidenziali scadono dopo quattro anni | Quando piove i lombrichi decollano con fatica |

**Table S.2**

*Filler sentences*

| **Subject-verb agreement (number)** | **Determiner-NP**  **(gender and number)** | **Verb and clitic** |
| --- | --- | --- |
| Proto-Theme | Gender | Gender |
| Nel bosco le volpi *scappa dal cacciatore  In cucina la torta *lievitano nel forno  Nella lavatrice la centrifuga *girano con grande rumore  In inverno la neve *cadono in montagna | Nel mare *le pesci nuotano liberi  In cucina *i pentole borbottano fumanti  Sugli spalti *le tifosi incitano la squadra  In laboratorio *gli operaie smontano la macchina | Il cane insegue il gatto e *la rincorre fin sotto il tavolo  Finito il libro, Sandro *la appoggia sulla libreria  Mario accende la radio e *lo sintonizza sul giornale locale  Il nonno raccoglie le patate e *li porta in cucina |
| Proto-Agent | Number | Number |
| In casa il pittore *dipingono le pareti  In estate le zanzare *punge le caviglie  Oggi la mamma *vanno al mercato  Di sabato i turisti *affolla il centro città | Al pomeriggio *il professori correggono i compiti degli alunni  Tutti i giorni *il ballerini si allenano per quattro ore  Oggi *la tartarughe nuotano libere nell’oceano  Nel pollaio *la galline depongono le uova al mattino | L’automobile sbanda e il camion *le urta  L’impiegata ritarda e il capoufficio *le chiama al telefono  Il vaso vacilla e Giovanni *li prende al volo  L’ape si avvicina e il turista *le scaccia |

**Table S.3**

*Quantitative description of target items (verbs).*

| **Verb variables** | **Correct sentences** | | **With violations** | | **value** | **p-value** |
| --- | --- | --- | --- | --- | --- | --- |
|  | **M** | **SD** | **M** | **SD** |  |  |
| Length | 7.50 | 1.93 | 7.48 | 1.96 | t = 0.01 | 0.97 |
| Log frequency | 4.48 | 5.10 | 4.48 | 5.10 | χ^2^ = 2548 | 0.24 |

*Note*. M = mean; SD = standard deviation.

**Data analyses on First Fixation Duration and Accuracy of responses**

**First Fixation Duration**

First Fixation Duration (FFD) is the duration in milliseconds of the first fixation on the target item and is considered as a proxy of early word processing (Liversedge et al., 1998)^^[[4]](#footnote-4)^^. Table S.4 and Figure S.1 summarize the mean durations (in milliseconds) of FFD on target verbs, by conditions, in the two groups. Table S.5 reports the estimated fixed parameters of the model, together with significance tests for the analysis on FFD.

**Table S.4**

*Mean duration in (ms) of FFD on target verbs*

|  | **HCs** | | | | **SSD** | | |  |
| --- | --- | --- | --- | --- | --- | --- | --- | --- |
|  | **Agent** | | **Theme** | | **Agent** | | **Theme** | |
|  | M | SEM | M | SEM | M | SEM | M | SEM |
| **Correct** | 261.93 | 3.36 | 266.88 | 3.69 | 310.79 | 5.29 | 313.18 | 4.92 |
| **With violation** | 274.72 | 3.75 | 268.63 | 3.74 | 319.50 | 5.72 | 314.63 | 5.18 |
| *Note***.** M = mean; SEM = Standard error of the mean | | | | | | | | |

**Figure S.1**

*Mean durations (in milliseconds) of FFD on target verbs, by conditions, in the two groups*


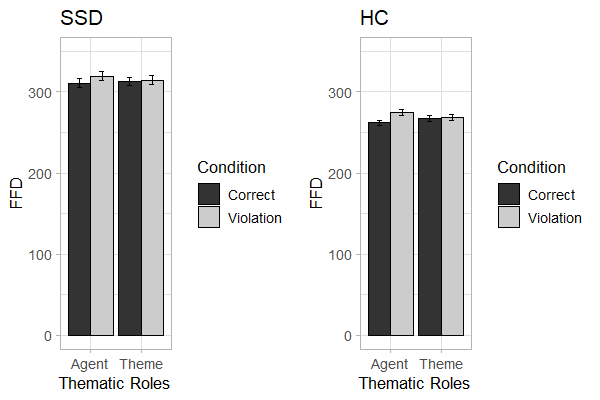


**Table S.5**

*Fixed and random effect on FFD*

| **Random effects** | **Variance** | **SD** |  |  |  |
| --- | --- | --- | --- | --- | --- |
| Subject (intercept) | .0201 | .1417 |  |  |  |
| Item (intercept) | .0003 | .0183 |  |  |  |
| Residual | .1015 | .3186 |  |  |  |
| **Fixed effects** | **Estimate** | **SD** | **df** | **t** | **p** |
| Intercept | 5.53 | .03 | 77.72 | 193.53 | < .001 |
| Condition (violated) | .03 | .02 | 5893.00 | 1.95 | .051 |
| Role (Theme) | .01 | .02 | 510.40 | .49 | .622 |
| Group (SSD) | .15 | .04 | 76.29 | 3.67 | < .001 |
| Condition * Role | -.04 | .02 | 5871.00 | -1.80 | .073 |
| Condition * Group | -.03 | .02 | 5849.00 | -1.08 | .279 |
| Role * Group | -.01 | .02 | 5848.00 | -.38 | .707 |
| Condition * Role * Group | .02 | .03 | 5847.00 | .76 | .450 |

Note. Df = Degrees of freedom; SD = Standard Deviation.

Of the three main effects considered, only that of *Group* (p < .001) was found to significantly affect FFD, suggesting that, irrespective of *Condition* and *Role*, participants with SSD show longer FFDs than HCs. Neither a two- nor a three-level interaction was found. Results can be explained bearing in mind that variables that may have affected FFDs, i.e. length and frequency of target verbs, had been controlled in the experimental material. For this reason, the significantly longer FFD for the SSD group cannot be imputed to this variable but rather to the general psychomotor delay that is generally observed in this group.

**Accuracy**

A score for Accuracy in the detection of violations was also computed as the percentage of correct responses. A response was considered correct if in agreement with the expected categorization (i.e., “acceptable” or “not acceptable”). As summarized in Table S.6, there were four possible answers that participants could produce:

1. they could categorize as acceptable a sentence that was actually correct (i.e., with no violation of the animacy trait of the subject, either a Theme or an Agent);
2. they could categorize as acceptable a sentence that contained a semantic violation of the subject (i.e., where the Agent or the Theme are not coherent with the verb);
3. they could categorize as non-acceptable a sentence that was actually correct; or
4. they could categorize as non-acceptable a sentence that contained a semantic violation of the subject.

In this study, we considered accurate only responses that fulfilled conditions (i) and (iv).

**Table S.6**

*Codification of possible answer*

|  | **Expected categorization** | |
| --- | --- | --- |
| **Participant responses** | **Acceptable** | **Not acceptable** |
| Acceptable | i. Correct | ii. Not correct |
| Not acceptable | iii. Not correct | iv. Correct |

Table S.7 and Figure S.2 report the accuracy by *Condition* and *Role* in the two groups. Table S.8 summarizes the estimated fixed parameters of the model for Accuracy, together with significance tests.

**Table S.7**

*Accuracy by Condition and Role in the two groups*

|  | **HC** | | **SSD** | |
| --- | --- | --- | --- | --- |
|  | **Agent** | **Theme** | **Agent** | **Theme** |
| **Correct** | 92% | 88% | 87% | 87% |
| **With violation** | 97% | 98% | 87% | 86% |

**Figure S.2**

*Accuracy by Group and Role*


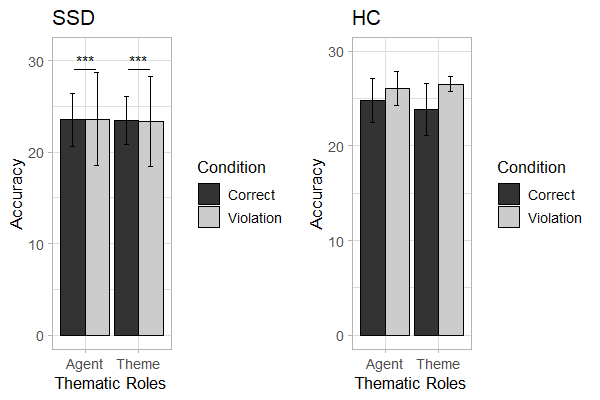


Note. *** significant at α ≤ .001.

**Table S.8**

*Fixed and random effect on accuracy*

| **Random effects** | **Variance** | **SD** |  |  |
| --- | --- | --- | --- | --- |
| Subject (intercept) | .7162 | .8463 |  |  |
| Item (intercept) | .3956 | .6289 |  |  |
| **Fixed effects** | **Estimate** | **SD** | **Z value** | **p** |
| Intercept | 2.79 | .24 | 11.50 | < .001 |
| Condition | 1.12 | .25 | 4.57 | < .001 |
| Role | -.40 | .25 | -1.62 | .106 |
| Group | -.52 | .29 | -1.80 | .072 |
| Condition * Role | 1.01 | .39 | 2.62 | .009 |
| Condition * Group | -1.04 | .29 | -3.56 | < .001 |
| Role * Group | .41 | .24 | 1.70 | .089 |
| Condition * Role * Group | -1.22 | .45 | -2.72 | .006 |

Note. SD = Standard Deviation.

A three-level interaction between fixed predictors was significant (z = -2.72, p < .01) with respect to accuracy scores. This result shows that *Condition* and *Role* acted differently in the two groups concerning explicit judgments.

Post-hoc comparisons indicate that HCs were significantly more accurate in categorizing sentences containing a violation than correct sentences, both concerning Agent (z = -4.57, p < .001) and Theme subjects (z = -7.09, p < .001).

On the contrary, the accuracy of responses of participants with SSD did not significantly differ between *Conditions*, neither on the Agent subject (z = -0.48, p = 1), nor on the Theme subject (z = 0.77, p = .992). This significant three-way interaction suggests an advantage for Theme-violation compared to Agent-violation sentences for HCs.

1. Paczynski, M., & Kuperberg, G. R. (2011). Electrophysiological evidence for use of the animacy hierarchy, but not thematic role assignment, during verb-argument processing. *Language and Cognitive Processes, 26*(9), 1402–1456. [↑](#footnote-ref-1)
2. Burzio, L. (1986). *Italian Syntax: a Government-Binding Approach* (Dordrecht, Springer);

   Perlmutter, D. M. (1978). Impersonal Passives and the Unaccusative Hypothesis. *Annual Meeting of the Berkeley Linguistics Society, 4*, 157. [↑](#footnote-ref-2)
3. Dowty, D. (1991). Thematic Proto-Roles and Argument Selection. *Language, 67*(3), 547;

   van Valin, R. D. (1997). Generalized semantic roles and the syntax-semantic interface. In *Empirical Issues in Formal Syntax and Semantics 2* (pp. 373–389). [↑](#footnote-ref-3)
4. Liversedge, S. P., Paterson, K. B., & Pickering, M. J. (1998). Eye movements and measures of reading time. In *Eye guidance in reading and scene perception* (pp. 55-75). Elsevier Science Ltd. [↑](#footnote-ref-4)
